# Hybrid imaging with [^68^Ga]PSMA-11 PET-CT and PET-MRI in biochemically recurrent prostate cancer

**DOI:** 10.1186/s40644-022-00489-9

**Published:** 2022-09-22

**Authors:** P. A. Glemser, L. T. Rotkopf, C. H. Ziener, B. Beuthien-Baumann, V. Weru, A. Kopp-Schneider, H. P. Schlemmer, A. Dimitrakopoulou-Strauss, C. Sachpekidis

**Affiliations:** 1 Department of Radiology, German Cancer Research Center (DKFZ), Heidelberg, Germany; 2 Medical Faculty, Ruprecht-Karls-University Heidelberg, 69120 Heidelberg, Germany; 3 Department of Biostatistics, German Cancer Research Center (DKFZ), Heidelberg, Germany; 4 Clinical Cooperation Unit Nuclear Medicine, German Cancer Research Center (DKFZ), Im Neuenheimer Feld 280, 69210 Heidelberg, Germany

**Keywords:** [^68^Ga]PSMA-11 ligand, PET-CT, PET-MRI, Prostate cancer recurrence, SUV, ADC

## Abstract

**Aim:**

To compare [^68^Ga]PSMA-11 PET-CT, [^68^Ga]PSMA-11 PET-MRI and MRI in a cohort of prostate cancer (PCa) patients in biochemical recurrence after initial curative therapy.

**Materials and methods:**

Fifty-three patients with biochemically recurrent PCa underwent whole-body [^68^Ga]PSMA-11 PET-CT 1 hour post-injection (p.i.) followed by [^68^Ga]PSMA-11 PET-MRI 2.5 hours p.i., including a multiparametric MRI pelvic protocol examination. Imaging data analysis consisted of visual (qualitative) evaluation of the PET-CT, PET-MRI and MRI scans, as well as semi-quantitative and quantitative analyses of the PET and MRI data, including calculation of the parameters standardized uptake value (SUV) and apparent diffusion coefficient (ADC) derived from the PCa lesions. Association analysis was performed between imaging and clinical data, including PSA level and Gleason score. The results were considered significant for *p*-values less than 0.05 (*p* < 0.05).

**Results:**

The hybrid imaging modalities [^68^Ga]PSMA-11 PET-CT and PET-MRI were positive in more patients than MRI alone. In particular, PET-CT detected lesions suggestive of PCa relapse in 34/53 (64.2%), PET-MRI in 36/53 (67.9%) and MRI in 23/53 patients (43.4%). While no significant differences in lesion detection rate were observed between PET-CT and PET-MRI, the latter was particularly efficient in detection of local recurrences in the prostate bed mainly due to the contribution of the MRI part of the modality. Association analysis revealed a statistically significant increase in the probability of a positive scan with increasing PSA levels for all imaging modalities. Accordingly, there was no significant association between scan positivity rate and Gleason score for any imaging modality. No significant correlation was observed between SUV and ADC values in lymph node metastases.

**Conclusion:**

[^68^Ga]PSMA-11 PET-CT and PET-MRI provide equally good detection rates for PCa recurrence, both outperforming stand-alone MRI.

## Introduction

The definition of biochemical recurrence of prostate cancer (PCa) is treatment-specific and has been a subject of debate. Although different cut-off values have been proposed, a PSA increase > 0.2 ng/ml after radical prostatectomy or an increase > 2 ng/ml from post-interventional PSA nadir following radiation therapy are widely applied [1, 2]. Biochemical recurrence can occur in approximately up to 40% of PCa patients and has a significant impact on progression to metastatic disease and patient mortality [3–5]. While the application of salvage radiotherapy of the prostate bed is recommended at low PSA levels (< 0.5 ng/ml) even without imaging guidance, the accurate localization of relapsing disease remains highly important for patient management [1]. Unfortunately, in PSA values < 10 ng/ml, PCa biochemical recurrence is often not associated with findings on conventional imaging modalities, such as CT or bone scan [6].

Recent developments in novel imaging modalities, such as multiparametric MRI (mpMRI) of the prostate as well as molecular imaging techniques have led to significant improvements in the evaluation of patients with biochemically recurrent PCa. In particular, mpMRI of the pelvis has been considered the cornerstone imaging modality in PCa patients with biochemical failure [7] being a highly accurate method for the identification of local recurrence, especially at low PSA levels (< 1.0 ng/ml) [8]. Moreover, in the last years, [^68^Ga]PSMA-11 PET-CT has emerged as an efficient diagnostic tool in PCa management especially in the context of biochemical recurrence, where it has rapidly become the investigation of choice [9–12]. However, the ligand [^68^Ga]PSMA-11 is rapidly renally excreted, leading to high radioactivity accumulation in the urinary bladder, which can, in turn, hamper evaluation of the prostate bed and even mask the detection of PCa local recurrence [13]. In this context, several methods have been developed to overcome this limitation, including early, dynamic [^68^Ga]PSMA-11 PET-CT acquisitions [14–17], the administration of diuretics [18–20] and the application of the novel radiotracer [^18^F]PSMA-1007, which is cleared mainly via the hepatobiliary excretion route [21, 22].

Another method that can potentially increase the diagnostic accuracy in biochemical recurrence of PCa is [^68^Ga]PSMA-11 PET-MRI, which combines the high performance of PSMA-PET for whole body assessment with the multiparametric potential and high soft tissue contrast of MRI, well suited to the locoregional evaluation of the prostate bed and pelvis. Although the initial results of the application of this novel imaging method have been promising [23–26], PET-MRI has not yet found its way in the clinical routine of PCa management.

Aim of this study was to compare the imaging modalities [^68^Ga]PSMA-11 PET-CT, [^68^Ga]PSMA-11 PET-MRI and MRI, including a mpMRI of the pelvis, in the evaluation of PCa patients presenting with biochemical recurrence after administration of initial curative therapy.

## Materials and methods

### Patients

Between June 2015 and November 2017 a total of 78 patients with biochemical recurrence of PCa after curative treatment underwent whole-body [^68^Ga]PSMA-11 PET-CT 1 hour post-injection (p.i.) followed by [^68^Ga]PSMA-11 PET-MRI approximately 2.5 hours p.i., including a mpMRI pelvic protocol examination, in our institute. Twenty-two patients from the initial cohort were excluded due to the usage of a PET-MRI protocol that was inconsistent with the planned study, two patients due to prior chemotherapy, and one patient due to lack of the exact PSA value at the time of the examinations, leaving a total cohort of 53 patients. Detailed patient characteristics are presented in Table 2. The study was approved by the ethics committee of the University of Heidelberg (S225/2021) and conducted in accordance to the declaration of Helsinki in its current form.

### Imaging data acquisition

#### PET-CT

Patients underwent a whole-body PET-CT 60 min after intravenous administration of [^68^Ga]PSMA-11. Imaging was performed from the head to the feet with an image duration of 2 min per bed position. A dedicated PET-CT system (Biograph mCT, S 128, Siemens Co., Erlangen, Germany) with an axial field of view of 21.6 cm with TruePoint and TrueV, operated in a three-dimensional mode was used. A low-dose attenuation CT scan (120 kV, 30 mA) was used for attenuation correction of the PET data and for image fusion. All PET images were attenuation-corrected and an image matrix of 400 × 400 pixels was used for iterative image reconstruction. Iterative image reconstruction was based on the ordered subset expectation maximization (OSEM) algorithm with two iterations and 21 subsets as well as time of flight (TOF) and point spread function (PSF).

#### PET-MRI

PET-MRI examinations were performed subsequently – as soon as possible - after the PET-CT studies, approximately 150 min after injection of [^68^Ga]PSMA-11. PET-MRI data was acquired using a dedicated PET-MRI system (3 T Biograph mMR, Siemens Healthineers, Erlangen, Germany). Given the prior time-consuming PET-CT procedure (total time from injection of the radiopharmaceutical to the end of the PET-CT scan approximately 90 minutes), a dedicated PET-MRI protocol of the abdomen and pelvis was applied in order to reduce patient discomfort. However, in the case of [^68^Ga]PSMA-11-positive lesions located in the head, neck or thorax regions, as identified in the initial PET-CT scan, the PET-MRI sequences were extended to the respective regions. PET and MRI data were acquired simultaneously. PET data (8 min per bed position, 6 min in pelvis bed position) were reconstructed with an iterative 3-D OSEM algorithm with two iterations, 21 subsets, 4 mm Gaussian filter and an image matrix 172, μ-map FOV. The acquisition protocol included a distinct pelvic MRI protocol (with high-resolution three-dimensional T2w, DWI with several b-values (b0, b50, b1000, b1500 s/mm^2^) and contrast-dynamic enhanced MRI) and additional morphological (T2w and CE-T1w) and diffusion-weighted sequences (b50 and b800 s/mm^2^) of the abdomen and pelvis [12, 23]. Detailed information to the applied MRI protocol is given below in Table 1.

**Table 1:** MRI protocol characteristics according to scanned body regions

	Sequence	Orientation	Slice thickness (mm)	TE (ms)	TR (ms)	Acquisition matrix	FOV (mm)	Flip Angle	Additional information
**Pelvic MRI protocol**	T2w TSE	Axial	3	146	8000	269 × 384	200 × 200	128°	
	T2w BLADE	Coronal	3	108	8110	320 × 320	260 × 260	126°	
	T2w BLADE	Sagittal	3	140	5470	256 × 320	200 × 200	130°	
	DWI (EPI Pelvic; b0, b50, b1000, b1500)	Axial	3	86	7500	96 × 128	210 × 280	90°	
	CE-T1w dynamic acquisition	Axial	2	2.12	4.5	176 × 256	275 × 400	15°	Number of acquisitions > 50, time spacing 5.6 sec, Gadovist® (body weight-adapted)
**Whole-body MRI protocol**	T2w HASTE	Coronal	6	41	800	186 × 256	717 × 501 (adapted)	91°	
	T2w HASTE	Axial	5	91	1000	194 × 320	315 × 420 (adapted)	136°	
	DWI (b50, b800)	Axial	5	59	6600	112 × 128	350 × 400 (adapted)	180°	
	CE-T1w VIBE fatsat	Axial	3	1.9	4.2	208 × 320	341 × 420 (adapted)	10°	
	CE-T1w VIBE fatsat	Coronal	3.5	1.2	3.9	192 × 352	677 × 420 (adapted)	10°	

*CE* Contrast-enhanced, *EPI* Echo-planar imaging, *TE* Time of echo, *TR* Time of repetition, *FOV* Field of view

### Imaging data analysis

#### Visual (qualitative) analysis

Visual analysis was performed by two board-certified radiologists (PAG, CHZ) and two board-certified nuclear medicine physicians (ADS, CS) without access to the patients’ clinical or laboratory data. The comparison of the different imaging modalities included only the body areas examined by all modalities in each patient. Discrepancies were resolved by consensus reading, which served as reference.

In particular, PET-CT image analysis was performed by the nuclear medicine physicians using a dedicated imaging software (aycan OsiriX^PRO^). Lesions with enhanced [^68^Ga]PSMA-11 uptake relative to local background were visually characterized as suspicious for PCa local recurrence or metastases after disregarding known benign [^68^Ga]PSMA-11 avid structures, such as ganglia or ureters.

With regard to PET-MRI, in an initial step the MRI-part of the examination was independently evaluated by the radiologists (syngo.via workstation, software version VB30, Siemens Healthineers, Erlangen, Germany) without access to the PET data. MRI lesions were classified using all available morphological and functional MRI sequences. In particular, the diagnosis of local recurrences was based on lesion shape, local invasiveness, restricted diffusion, central necrosis and increased contrast enhancement. Lymph nodes were considered as suspicious for metastatic involvement based on a short axis diameter of > 8 mm (pelvic) or > 10 mm (neck, thoracic, abdominal, inguinal), spherical configuration, irregular border, inhomogeneity, marked diffusion restriction and increased contrast enhancement. Respectively, as distant metastases were regarded those lesions that were detectable as locally invasive and showing pathological contrast enhancement, central necrosis or diffusion restriction [27, 28].

At a second step, the hybrid PET-MRI scans were evaluated interdisciplinary by each one of the above-mentioned nuclear medicine physicians (CS) and radiologists (PAG) under simultaneous consideration of both the PET and MRI parts of the examination. In order to reduce bias, the interdisciplinary reading of PET-MRI was performed at least 4 weeks after the initial reading sessions of PET-CT and the MRI part of PET-MRI. Similarly to PET-CT, PET lesions of the PET-MRI examination were classified as suspicious for PCa local recurrence or metastases based on an enhanced [^68^Ga]PSMA-11 uptake relative to local background, while also taking into consideration the respective findings in the morphological and functional MRI sequences.

#### Semi-quantitative and quantitative analysis

##### Number of suspicious lesions

The number of PCa-suspicious lesions was determined in each modality, with a maximum of up to 20 measured lesions per patient; in patients with disseminated metastases (> 20 lesions), by definition the lesions were classified as uncountable and were excluded from the lesion-based statistical analysis.

##### Standard uptake value (SUV)

The semi-quantitative PET evaluations were based on volumes of interest (VOIs) placed over tumor-suspicious lesions and the subsequent calculation of SUVmean and SUVmax (PMOD Technologies Ltd, Zürich, Switzerland) [29]. In patients with disseminated metastases, calculations were limited to the five most visually prominent lesions.

##### Apparent diffusion coefficient (ADC)

ADC values were determined using a dedicated, open source software application (ITK-SNAP, http://www.itksnap.org) by manually segmenting each lesion in the ADC-map [30]. Smaller lesions not easily visible in the ADC-map were segmented in the high b-value-image and then transferred to the respective ADC-map.

### Statistical analysis

Difference in the imaging modalities was assessed with regard to the number of lesions. Specifically, to account for measurements on the same patient with different modalities, a Poisson mixed effects model was fit with fixed factor for image modality and random intercept for the patient. For each imaging modality, patients were classified into either positive status or negative status depending on the presence or absence of PCa lesions. Further grouping of patients was done based on the PSA levels as follows; Group A: ≤0.2 ng/ml, Group B: > 0.2 and ≤ 0.5 ng/ml, Group C: > 0.5 and ≤ 2.0 ng/ml, and Group D: > 2.0 ng/ml. Association between presence/absence of lesions and PSA levels was investigated using Cochran-Armitage test for trend for each imaging modality. To investigate the association between presence/absence of lesions with the different imaging modalities and Gleason score, patients were grouped based on their Gleason score. The Cochran-Armitage test for trend was then applied to determine whether there was a statistically significant trend. Correlation between the SUV and ADC parameters was determined using a mixed-effects model approach as proposed by Hamlett et al. [31] because of the presence of repeated measurements for subjects. Here, we use the log-transformed SUV and ADC parameters as the original variables were skewed. Confidence intervals for the correlation estimate were obtained using a normal approximation as in Irimata et al. [32]. Correlation between the log-transformed SUV and ADC parameters was also performed in a subcohort of patients who had lesions located in the lymph nodes and was further divided into four groups based on their short axis diameter (Group 1: < 0.4 cm, Group 2: ≥0.4 and < 0.7 cm, Group 3 ≥ 0.7 and < 1 cm, and Group 4: ≥1 cm). The analysis was performed with dedicated statistical software programs (R version 4.0.3, package lme4; SAS 9.4). The results were considered significant for *p*-values less than 0.05 (*p* < 0.05).

## Results

### Patient-based analysis

The included patients had a median PSA level of 1.60 ng/ml (range = 0.07–25.9 ng/ml). The Gleason Score was available in 41/53 patients (77.4%) and its median value was 7 (range = 6–9). After exclusion of the two patients with innumerable lesions (> 20), in total 108 lesions were detected by PET-CT, 109 lesions by PET-MRI, and 35 lesions by MRI.

**Fig. 1:**
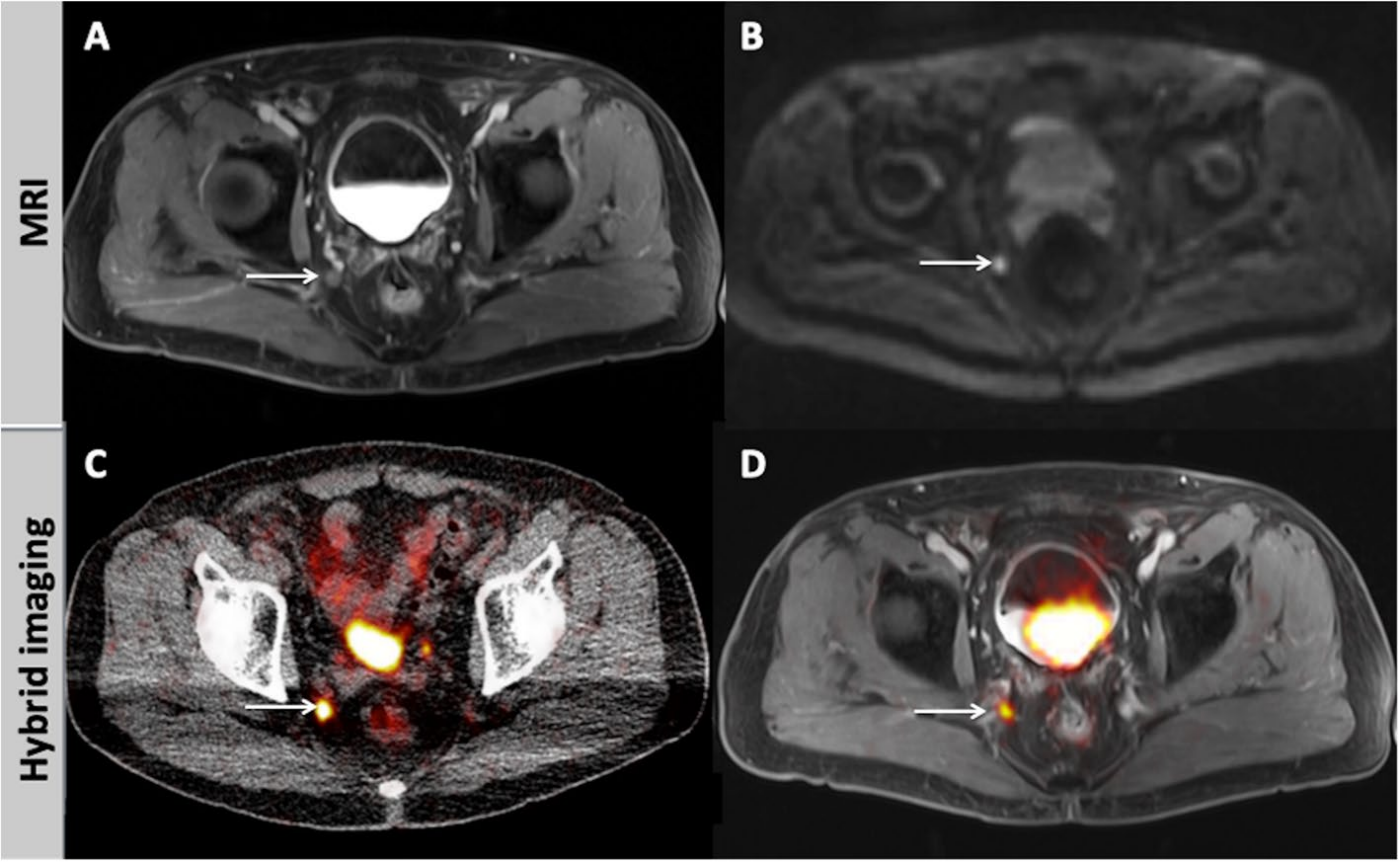
A 75-year old patient with biochemical recurrence of PCa referred to our institution for imaging. His PSA level at the time of examination was 4.1 ng/ml. Contrast enhanced-T1w fatsat (**A**) and DWI (b800; **B**) at the pelvic level show a 9 mm, rounded, right obturator lymph node with irregularly spiculated border and heterogeneous contrast agent enhancement (arrow, **A**) and distinct diffusion restriction with signal increase in the b800 image (arrow, **B**). The lymph node also exhibited signal decrease in the corresponding ADC map (not shown). In the fused hybrid modalities PET-CT (**C**) and PET-MRI (**D**), the lymph node also shows a focal, intensive [^68^Ga]PSMA-11 uptake, suggestive of metastatic involvement (arrow)

**Fig. 2:**
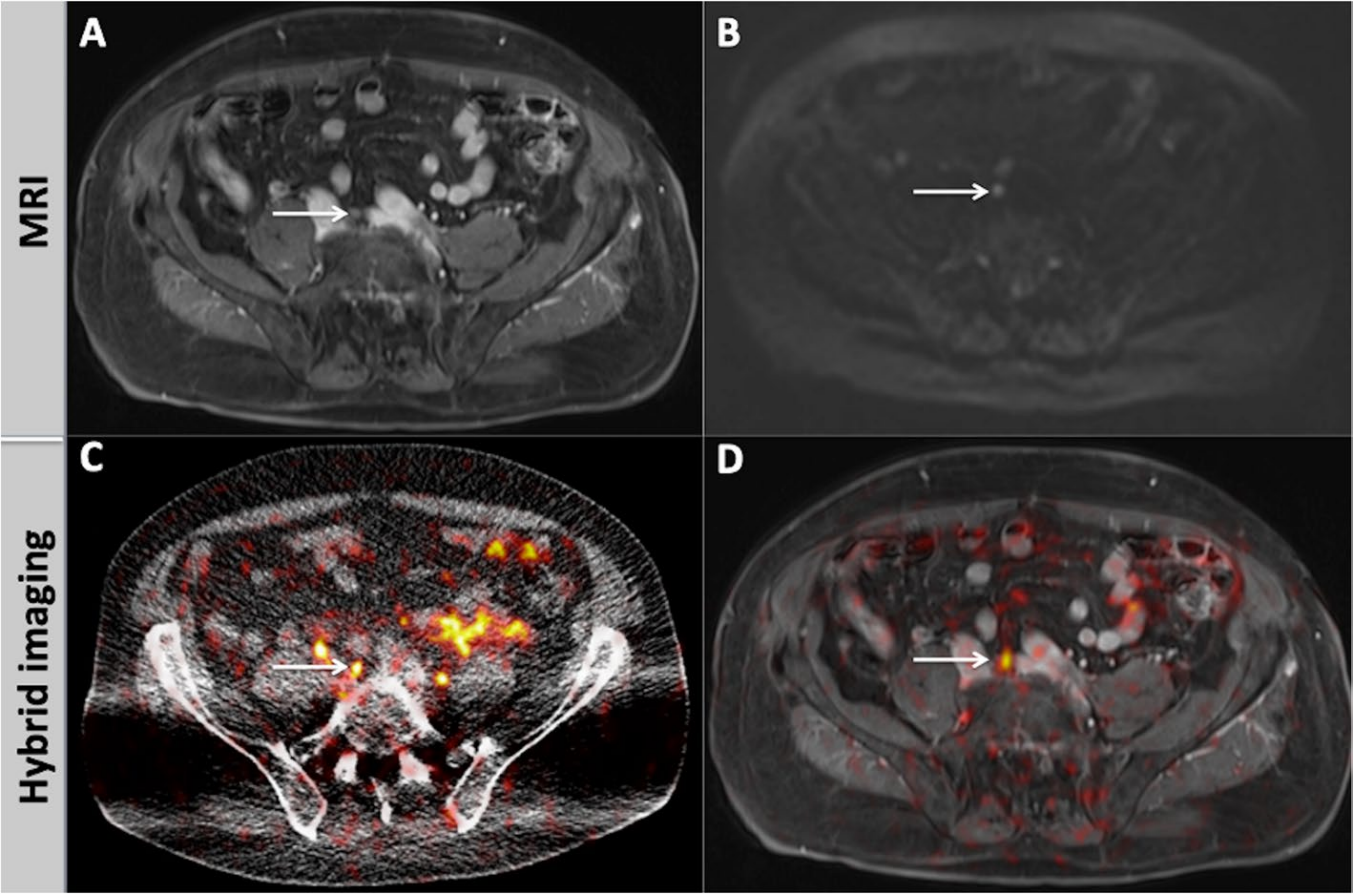
A 76-year old patient with biochemical recurrence of PCa referred to our institution for imaging. His PSA level at the time of examination was 5.0 ng/ml. Contrast enhanced-T1w fatsat (**A**) and DWI (b800; **B**) at the pelvic level merely show a small (5 mm), oval, presacral lymph node (arrow), which was not considered suspicious. In the fused hybrid modalities PET-CT (**C**) and PET-MRI (**D**), however, the above-mentioned lymph node shows a focal, intensive [^68^Ga]PSMA-11 uptake, suggestive of metastatic involvement (arrow). In PET-CT (**C**), both ureters are visible laterally of the mentioned lymph node

**Fig. 3:**
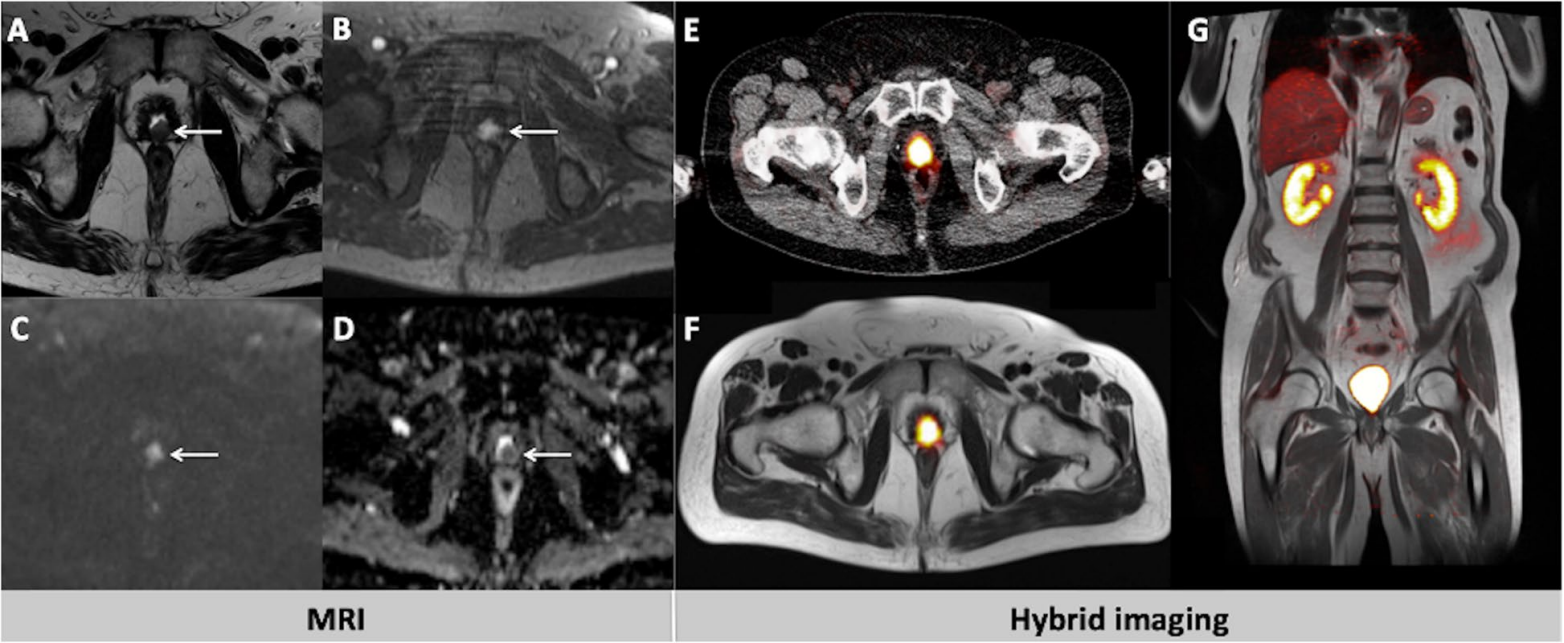
A 77-year old patient with biochemical recurrence of PCa after radical prostatectomy. His PSA level at the time of examination was 2.0 ng/ml. In MRI, a lesion in the prostate bed with hypointense T2w signal, suspicious for PCa local recurrence is depicted (**A**, arrow). The lesion shows marked early contrast enhancement in the dynamic T1w (**B**, arrow) and distinct diffusion restriction with signal increase in the b1500 image (**C**, arrow) and corresponding signal decrease in ADC map (**D**, arrow). In the hybrid imaging modalities PET-CT (**E**) and PET-MRI (**F**, **G**), the suspicious lesion is overseen due to its masking by intensive tracer accumulation in the urinary bladder

**Fig. 4:**
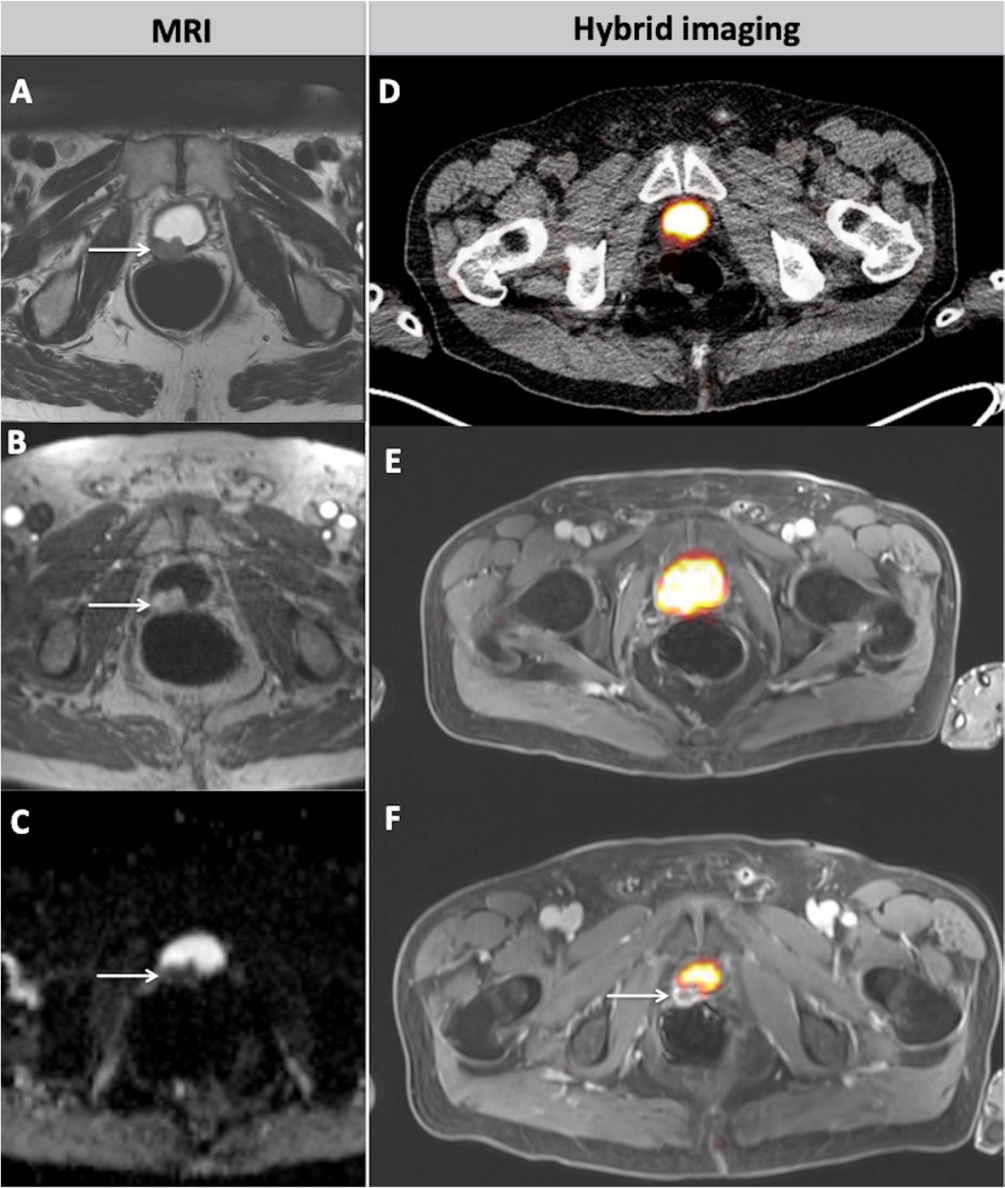
A 66-year old patient referred for imaging due to biochemical failure of PCa after radical prostatectomy and ADT. The patient presented with a PSA level of 1.2 ng/ml. In MRI, a lesion in the prostate bed with hypointense to intermediate T2w signal, suspicious for PCa local recurrence is depicted (**A**, arrow). The lesion shows early contrast enhancement in the dynamic T1w (**B**, arrow) and distinct diffusion restriction with signal decrease in ADC map (**C**, arrow). However, in PET-CT (**D**) no pathological tracer uptake can be delineated. In PET-MRI, the cranial part of the local recurrence is masked by the physiological [^68^Ga]PSMA-11 accumulation in the urinary bladder (**E**), while its caudal part can be clearly delineated as a contrast enhancing lesion in the MRI part of the fused image (arrow, **F**), despite the lack in [^68^Ga]PSMA-11 uptake

**Fig. 5:**
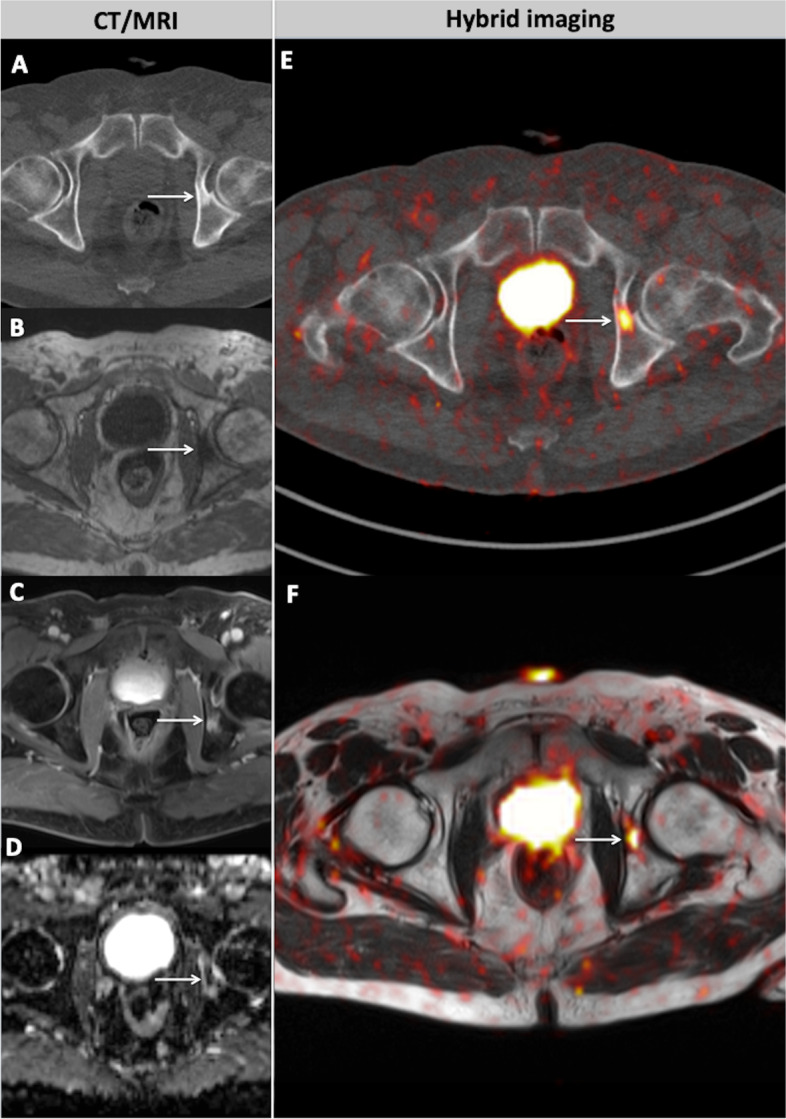
A 74-year old patient with biochemical recurrence of PCa after radical prostatectomy (PSA = 1.68 ng/ml). The patient showed a sclerotic lesion in the left acetabulum as a sign of an osseous metastasis in CT (**A**, arrow). In MRI, correspondingly, the lesion appears hypointense in T1w (**B**, arrow) with contrast enhancement (**C**, arrow) and signal decrease in ADC map of the DWI (**D**, arrow). Respectively, both hybrid imaging modalities PET-CT (**E**, arrow) and PET-MRI (**F**, arrow) depict the osseous lesion as a focus of markedly increased [^68^Ga]PSMA-11 uptake, highly suggestive of metastasis

PET-CT revealed suspicious lesions in 34/53 patients (64.2%), while PET-MRI was positive in 36/53 patients (67.9%). The MRI-part of PET-MRI was positive in 23/53 patients (43.4%). Hybrid imaging modalities (PET-CT, PET-MRI) were positive in more patients than MRI alone (*p* < 0.05). On the other hand, no significant differences were observed between PET-CT and PET-MRI regarding presence of lesions on a patient basis. Patient characteristics, number of lesions, distribution of these lesions as well as potential treatment changes based on the PET-MRI findings, as compared to PET-CT, are depicted in Table 2. Figures 1, 2, 3, 4 and 5 present examples of the studied patients.

**Table 2:** Characteristics of the enrolled patients

Patient number	Age (years)	PSA at examination (ng/ml)	Previous treatment	Gleason Score	Injected activity (MBq)	Number of lesions (Localisation)		
						PET-CT	PET-MRI	MRI
1	59	0.6	RPx	n.a.	144	0	0^a^	0
2	79	4.8	RPx + RT + ADT	9	145	2 (LN)	2 (LN)^a^	0
3	59	7.18	RT	7	141	1 (LR)	1 (LR)^a^	0
4	76	4.95	HIFU + ADT	9	105	2 (LR, LN)	1 (LR)^b^	1 (LR)
5	62	0.39	RPx	8	121	1 (LN)	1 (LN)^a^	0
6	74	4.2	RPx	n.a.	235	> 20 (LN, B)	> 20 (LN, B)^a^	9 (LN)
7	56	2.4	RPx	7	264	0	1 (O)^c^	1 (O)
8	73	0.68	RPx	7	252	1 (LN)	0^c^	0
9	59	0.2	RPx	n.a.	265	0	0^a^	0
10	71	1.7	RPx	7	107	10 (LN)	14 (LN)^b^	6 (LN)
11	67	0.58	RPx + RT	7	98	2 (LN)	1 (LN)^b^	0
12	79	0.46	RPx	9	255	0	0^a^	0
13	53	2.9	RPx + RT + ADT	9	214	1 (B)	1 (B)^a^	0
14	75	4.3	RPx + RT + ADT	n.a.	242	5 (LN)	5 (LN)^a^	0
15	77	2.0	RPx	7	135	0	1 (O)^c^	1 (O)
16	61	0.45	RPx	7	199	0	0^a^	0
17	66	10.0	Electrocoagulation of the prostate	7	250	> 20 (LN)	> 20 (LR, LN)^b^	8 (LR, LN)
18	71	0.39	RPx + RT	7	252	0	0^a^	0
19	66	1.2	RPx + ADT	n.a.	165	0	1 (LR)^c^	1 (LR)
20	64	4.5	RPx + RT	8	165	0	0^a^	0
21	68	1.2	RT + ADT	9	154	1 (LR)	1 (LR)^a^	1 (LR)
22	69	4.73	RPx	8	150	1 (LN)	1 (LN)^a^	1 (LN)
23	74	1.68	RPx	7	140	2 (B)	2 (B)^a^	2 (B)
24	70	1.5	RPx + RT	7	196	8 (LN, B)	8 (LN, B)^a^	2 (LN, B)
25	59	1.15	RPx + RT	6	172	1 (LN)	1 (LN)^a^	0
26	77	0.29	RPx + RT + ADT	9	224	7 (LN)	7 (LN)^a^	2 (LN)
27	75	0.2	RPx	7	228	1 (LN)	0^c^	0
28	68	1.18	RPx + RT	7	211	0	0^a^	0
29	70	25.9	RPx + ADT	7	199	18 (LN)	18 (LN)^a^	3 (LN)
30	56	2.0	RPx + RT	n.a.	197	1 (LN)	1 (LN)^a^	0
31	66	3.9	RPx + RT + ADT	n.a.	272	1 (LN)	1 (LN)^a^	0
32	81	0.8	RPx + RT	7	149	1 (B)	1 (B)^a^	0
33	75	4.1	RT	n.a.	222	3 (LN)	3 (LN)^a^	2 (LN)
34	77	2.02	RPx + ADT	9	287	5 (LN)	3 (LR, LN)^b^	1 (LR)
35	69	7.1	RPx + RT + ADT	n.a.	144	13 (LN)	13 (LN)^a^	0
36	70	2.8	RPx + RT + ADT	n.a.	235	3 (LN)	3 (LN)^a^	2 (LN)
37	49	1.73	RPx	7	141	1 (LR)	1 (LR)^a^	1 (LR)
38	63	1.6	RPx	n.a.	117	0	1 (LN)^c^	1 (LN)
39	69	12.15	RPx	7	199	0	0^a^	0
40	71	0.35	RPx	7	217	0	0^a^	0
41	71	0.54	RPx + RT	7	259	0	0^a^	0
42	69	3.6	RPx	n.a.	198	3 (LN)	2 (LN)^b^	1 (LN)
43	64	0.15	RPx	7	71	0	0^a^	0
44	61	1.0	RPx	9	180	1 (LN)	1 (LN)^a^	1 (LN)
45	56	0.67	RPx	7	240	1 (LN)	2 (LN)^b^	2 (LN)
46	66	6.2	RPx + ADT	9	247	1 (LN)	1 (LN)^a^	1 (LN)
47	70	0.07	RPx	8	238	0	0^a^	0
48	60	1.09	RPx + RT	8	166	0	0^a^	0
49	71	0.2	RPx	7	250	0	0^a^	0
50	74	0.31	RPx + RT	8	256	0	0^a^	0
51	67	3.5	RPx + ADT	7	156	5 (LN)	4 (LN)^b^	0
52	60	0.7	RPx	7	143	4 (LN)	4 (LN)^a^	2 (LN)
53	79	1.76	RPx + RT	7	170	1 (B)	1 (B)^a^	0

*n.a.* Not available, *RPx* Radical prostatectomy, *RT* Radiotherapy, *ADT* Androgen deprivation therapy, *HIFU* High-intensity focused ultrasound, *LN* Lymph node metastasis, *LR* Local recurrence, *B* Bone metastasis, *O* Other organ metastases

^a^Patients with concordant findings in PET/MRI and PET/CT

^b^Patients with vastly concordant findings in PET/MRI and PET/CT that would potentially lead to no treatment changes based on the different imaging results

^c^Patients with significant differences between PET/MRI and PET/CT that would potentially lead to treatment changes based on the different imaging results

### Association between clinical parameters and imaging findings

#### PSA plasma levels

**Fig. 6:**
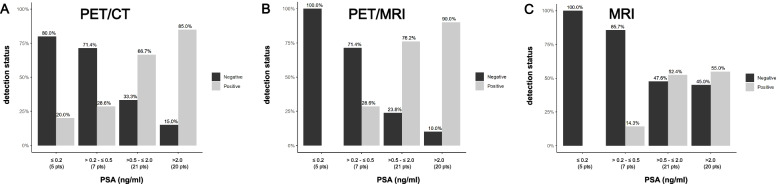
Comparison of the positivity rate of the different imaging modalities after classification of patients in groups based on their PSA plasma levels. Patients were separated in four groups: Group A, PSA ≤0.2 ng/ml; Group B, PSA > 0.2 and ≤ 0.5 ng/ml; Group C, PSA > 0.5 and ≤ 2.0 ng/ml; Group D, PSA > 2.0 ng/ml. A statistically significant increase in the positivity rate in all imaging modalities (PET-CT, PET-MRI, MRI) with increasing PSA levels is observed (**A**, **B**, **C**)

After patient classification in four groups, based on their plasma PSA levels, association analysis showed a statistically significant increase in the probability of a positive imaging scan (PET-CT, PET-MRI, MRI) with increasing PSA levels (*p* < 0.05) (Fig. 6).

#### Gleason score

**Fig. 7:**
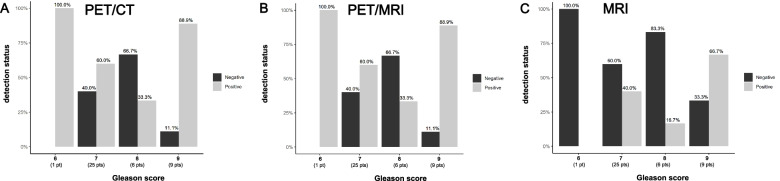
Comparison of the positivity rate of the different imaging modalities after patient classification in groups based on their Gleason Score. Patients were separated in four groups by Gleason score (6–9). The probability of a positive examination does not seem to significantly increase as Gleason score increases for any imaging modality (**A**, **B**, **C**)

After patient classification in four groups, based on their Gleason score, association analysis showed no statistically significant increase of the examination positivity rate with an increase of Gleason score for any imaging modality (Fig. 7).

### Lesion-based analysis

Two patients demonstrated disseminated metastatic disease (> 20 lesions) in hybrid imaging modalities, and were, thus, excluded from the lesion-based analysis. In the remaining 51 patients, the median number of positive lesions by modality was 1 in PET-CT (range 0–18), 1 in PET-MRI (range 0–18), and 0 in the MRI part of PET-MRI (range 0–6). Hybrid imaging modalities demonstrated a significantly higher number of lesions compared with MRI alone (*p* < 0.0001). On the other hand, no significant differences were noted between PET-CT and PET-MRI.

#### Semiquantitative and quantitative analysis

With regard to PET-CT, the average SUVmean of the evaluated PCa lesions was 12.9 (median = 9.6) and the average SUVmax was 20.9 (median = 15.4). Respectively, in PET-MRI, the average SUVmean was 10.6 (median = 7.4) and the average SUVmax was 15.8 (median = 10.9). In MRI, the average ADCmean value was 821.6 μm^2^/s (median = 834.3 μm^2^/s). Statistical analysis regarding lymph node metastases showed no significant correlation between the log-transformed SUV values (SUVmean, SUVmax) and ADCmean both in the overall cohort (irrespective of the lymph nodes’ size) as well as under consideration of lymph node subgroups based on their size (Group 1: < 0.4 cm, Group 2: ≥0.4 and < 0.7 cm, Group 3 ≥ 0.7 and < 1 cm, and Group 4: ≥1 cm).

## Discussion

In an attempt to further investigate the phenomenon of PCa biochemical recurrence, we retrospectively compared the imaging modalities [^68^Ga]PSMA-11 PET-CT, PET-MRI and MRI in a cohort of 53 PCa patients. Our results showed, firstly, that the hybrid modalities PET-CT and PET-MRI provided equally good detection rates of PCa recurrence, both outperforming stand-alone MRI. Secondly, we could confirm the positive association between PSA plasma levels and detection rate of PCa lesions by hybrid imaging modalities. On the other hand, no such association was observed with regard to Gleason-Score. Finally, no significant correlation between the quantitative parameters SUV and ADC was observed in lymph node metastases.

The comparison of PET-CT with PET-MRI showed an equal performance for the two modalities with very similar detection rates of suspicious PCa lesions (64.2% vs. 67.9% patient-based positivity rate, respectively). This finding is in line with the results of a recent meta-analysis, including 5 studies with 219 patients, which evaluated the diagnostic performance of [^68^Ga]PSMA-11 PET/CT vs. PET/MRI for biochemical recurrence of PCa and demonstrated an equivalent performance for the two methods [33]. In total, in our study PET-CT revealed 8 lesions (7 patients) not detectable with PET-MRI. This difference can be attributed to the protocol applied in the present study, with PET-MRI being performed approximately 150 min p.i. and after the completion of PET-CT. A second administration of [^68^Ga]PSMA-11 at a different time-point (for instance 1 day after the initial application of the radiotracer for the PET-CT study) in terms of the PET-MRI examination could not be justified for radiation protection reasons. However, the performance of PET-MRI at a - similar to PET-CT - earlier time-point, i.e. 60 min p.i., would have presumably led to detection of more lesions, potentially minimizing the difference between the modalities. This explanation is further supported by the dedicated PET-MRI protocol applied in our study, rendering the intermodal (PET-CT vs. PET-MRI) attenuation correction differences rather unlikely as responsible for this difference.

On the other hand, PET-MRI also detected some lesions (7 patients) not seen with PET-CT. These discordant findings were attributed either to local recurrences visible only on the MRI part of the examination due to masking through radiotracer accumulation in the bladder (*n* = 3 patients) or to PSMA-negative lesions in the lymphatic (*n* = 3 patients) and pulmonary system (*n* = 1 patient). This is in accordance with previous results of other groups, who also highlighted the equal performance of PSMA-based PET-CT and PET-MRI in PCa detection with a slight superiority of the latter modality in detection of local recurrences [23–26]. In particular, Freitag et al. reported similar accuracy and reliability for [^68^Ga]PSMA-11 PET-CT and PET-MRI, acquired at 1 h and 3 h p.i. respectively, in the detection of lymph node and bone metastases. In line with our results, the authors could demonstrate the value of both hybrid imaging techniques in depicting „normal-sized “lymph nodes as suspicious due to increased PSMA uptake with a high intermodal concordance of 98.5% [24]. Further, Guberina et al. could reveal the detection of 11 additional lesions with PET-MRI compared with PET-CT (6 local recurrences, 5 lymph nodes) in a cohort of 93 patients, leading to different treatment advices in those patients with local recurrences [25]. In 4/53 patients of our cohort, PET-MRI revealed more lesions than PET-CT that would have potentially led to an alteration in treatment (Table 2). However, given the retrospective nature of our study and the lack of follow-up data, such management changes could not be verified. In a recent prospective study, involving 34 patients with biochemically recurrent PCa, the incremental value of [^68^Ga]PSMA-11 PET-MRI versus PET-CT was also investigated. It was shown that all lesions detected on PET-CT were also detected on PET-MRI. In addition, more lesions were depicted on PET-MRI than on PET-CT (88 vs. 81). The authors concluded that PET-MRI is able to detect biochemically recurrent PCa at least as accurately as PET-CT for local recurrence, lymph node metastasis and distant metastasis, while the substitution of PET-CT by PET-MRI adds sensitivity in PSMA lesion detection also in the setting of distant recurrence due to both the MR and TOF PET components [26].

With regard to the intermodal comparison of PET-hybrid modalities versus MRI, the former exhibited a significantly higher detection rate both in patient- and lesion-based analysis, which was especially pronounced in detection of lymph node and bone metastases. These findings support prior results comparing PSMA-based hybrid modalities with MRI (whole-body or multiparametric MRI of the pelvis), which, likewise, demonstrated the superiority of hybrid imaging in the detection of PCa lesions in the lymphatic and skeletal system. In particular, in a study of 28 PCa recurrent patients with low - comparable to our cohort - PSA serum levels, Sawicki et al. found that whole-body MRI was inferior to PET-CT in detection of subcentimetre but PSMA-avid nodal metastases, which were considered benign according to radiological criteria [28]. Further, Rauscher et al. revealed a distinct difference between PSMA PET and morphologic imaging for detection of lymph node metastases in histologically proven lymph node fields/regions (77.9% versus 26.9%) [34]. These results were substantiated by Asfhar-Oromieh et al., who compared PSMA-ligand PET/CT with multiparametric MRI and reported the detection of 32 certain lymph node metastases in PET versus 12 in MRI in a cohort of 43 patients [35]. Moreover, in a prospective comparison of the diagnostic accuracies of [^68^Ga]PSMA PET-CT and diffusion-weighted MRI in 68 PCa patients with biochemical recurrence, the former demonstrated a significantly higher diagnostic performance for detection of bone metastases [36]. Recently, in an observational, comparative study of [^68^Ga]PSMA PET-MRI versus conventional mpMRI in a population of patients with biochemically recurrent PCa (*n* = 114 patients), hybrid imaging had a significantly higher detection rate and sensitivity than mpMRI [37]. Finally, in the framework of another approach, in a cohort of 41 patients with locally recurrent PCa after primary radiotherapy, the combined use of [^68^Ga]PSMA-11 PET-CT and mpMRI led to a positive predictive value of 97.6% for detecting recurrent PCa, suggesting that targeted biopsies can be safely withheld from the workup towards focal salvage high dose rate brachytherapy, provided that the results of the imaging methods are conclusive [38].

Apart from the detection of nodal metastases, special focus was placed in the detection of local PCa lesions in the prostate bed, since this is one of the most relevant localizations in the clinical context of early recurrence, as manifested by low but increasing PSA levels after curative treatment. Considering the well-known limitations of [^68^Ga]PSMA-11 PET-CT in detecting tumors in this anatomical region [13], we investigated the potential role of PET-MRI in delineation of such lesions. Our results showed that 3/7 PCa local recurrences were negative in PET imaging and could only be exhibited in the MRI part of the PET-MRI examination. Respectively, 3/7 PCa local recurrences could be delineated as [^68^Ga]PSMA-11-avid lesions in both hybrid modalities, while one lesion in the prostate bed could only be found in PET-CT. These findings support the application of a distinct multiparametric pelvic MRI protocol performed in addition to whole-body PET imaging – optimally in the context of a combined PET-MRI study - in order to enhance the diagnostic accuracy in PCa recurrence detection [25, 26, 39]. On the other hand, PET-MRI carries some significant limitations, which restrict its broad application, including high costs, logistical issues derived from the longer acquisition protocols, the need for multidisciplinary (nuclear medicine and radiology) reading and analysis of patient data, patient devices not compatible with MR-imaging (i.e. pacemaker) and potential patient discomfort. However, some of these problems may be satisfyingly remedied by introduction of some recently proposed, modified, faster PET-MRI acquisition protocols [40] or modified combination protocols of PET-CT and PET-MRI [41].

We also studied the association between the positivity of imaging modalities and clinically relevant parameters for PCa. Our results revealed a statistically significant, positive trend between higher PSA levels and detection of lesions by both hybrid modalities, which is in line with previously reported findings with PSMA-ligand PET-CT [9, 10].

Further, the potential association between positivity of imaging modalities and Gleason score was investigated. Thereby no significant association was observed irrespective of the applied imaging modality. Previous studies addressing the correlation between PSMA PET and Gleason score in the PCa recurrence setting have been so far inconclusive with some groups reporting a significantly positive correlation [42], while others not [9, 43].

Finally, we explored the potential correlation between the quantitative parameters SUV, derived from [^68^Ga]PSMA-11 PET-CT and PET-MRI, and the corresponding ADC values in lymph nodes metastases, the most frequently detected lesions in our series. No significant correlation was observed between SUV and ADC values both in the whole cohort and in separate lymph node groups, classified according to their size. The reason for this lack of correlation may lie in the different molecular mechanisms reflected by each parameter with ADC being an imaging marker of cellularity, while SUV expresses the intensity of PSMA expression in PCa cells. In addition, it cannot be excluded that the expression of a cell membrane protein/receptor can be macroscopically averaged as high uptake, i.e. high SUV, from not very cell dense stroma, while low SUV values can be read from packed cells with very low receptor expression. Further, the effect of prior treatments, including radiotherapy and/or ADT, on these functional parameters cannot be assessed. To our knowledge these are the first published results regarding the correlation between ADC and SUV values in PSMA PET hybrid imaging in the context of PCa biochemical recurrence. Using [^18^F]-choline PET-MRI Piccardo et al. reported on an inverse correlation between SUVmax and ADC values in PCa local recurrences and lymph nodes metastases in a cohort of 21 patients with biochemically recurrent PCa after radiotherapy [44]. However, our results are not comparable with the aforementioned study due to the different radiotracers applied, with [^18^F]-choline being a marker of phospholipid turnover, and [^68^Ga]PSMA-11 reflecting expression of the transmembrane protein PSMA.

We note some limitations in our study. Firstly, the number of patients included is relatively small, which is however, mainly attributed to the extended acquisition protocol followed. Secondly, due to the retrospective nature of the study, some information regarding patient and disease characteristics are incomplete, including surgical margins, local invasion and Gleason Score. Thirdly, the extension of dedicated PET-MRI sequences in the head-thorax region was triggered by positive findings on PET/CT, which differs from the regular, whole-body integrated PET-MRI acquisition protocols; this approach may lead to possible bias in calculating imaging detection rate and accuracy in identifying metastatic lesions. Further, the lack of definitive pathologic correlation or confirmatory imaging follow-up that would serve as standard of reference for the identified findings constitute further limitations. Therefore, a validation of these findings in prospectively conducted studies including larger patient cohorts would be beneficial. Finally, the vast majority of the PCa-suggestive imaging findings was not histopathologically confirmed, which is usually not done in the clinical setting.

## Conclusions

This study aimed to compare the imaging modalities [^68^Ga]PSMA-11 PET-CT, [^68^Ga]PSMA-11 PET-MRI and MRI in a cohort of PCa patients in biochemical recurrence after initial curative therapy. Our results revealed equally good tumor detection rates for the hybrid imaging modalities PET-MRI and PET-CT, both outperforming stand-alone MRI. [^68^Ga]PSMA-11 PET-MRI was particularly efficient in the detection of local PCa recurrences mainly due to the contribution of the mpMRI part of the modality. Based on this, the application of [^68^Ga]PSMA-11 PET-CT followed by subsequent PET-MRI, or alternatively the combination of [^68^Ga]PSMA-11 PET-CT with multiparametric pelvic MRI represent appropriate imaging protocols for improving PCa recurrence detection.

## Data Availability

The datasets generated during and/or analysed during the current study are available from the corresponding author on reasonable request.

## Abbreviations

[^18^F]-choline: ^18^fluoride choline
[^18^F] PSMA-1007: ^18^fluoride prostate-specific membrane antigen 1007
[^68^Ga] PSMA-11: ^68^gallium prostate-specific membrane antigen 11
ADC: Apparent diffusion coefficient
CE-T1w: Contrast-enhanced T1 weighted (image)
CT: Computed tomography
DWI: Diffusion-weighted imaging
FOV: Field of view
mpMRI: Multiparametric magnetic resonance imaging
MR: Magnetic resonance
MRI: Magnetic resonance imaging
OSEM: Ordered subset expectation maximization
p.i.: Post-injection
PCa: Prostate cancer
PET: Positron emission tomography
PET-CT: Positron emission tomography-computed tomography
PET-MRI: Positron emission tomography–magnetic resonance imaging
PSA: Prostate-specific antigen
PSF: Point spread function
PSMA-PET: Prostate-specific membrane antigen positron emission tomography
SUV: Standardized uptake value
T1w: T1-weighted (image)
T2w: T2-weighted (image)
TOF: Time of flight
VOI: Volume of interest
